# Association of ATG4B and Phosphorylated ATG4B Proteins with Tumorigenesis and Prognosis in Oral Squamous Cell Carcinoma

**DOI:** 10.3390/cancers11121854

**Published:** 2019-11-23

**Authors:** Pei-Feng Liu, Hung-Chih Chen, Jin-Shiung Cheng, Wei-Lun Tsai, Huai-Pao Lee, Shu-Chi Wang, Wei-Hao Peng, Cheng-Hsin Lee, Luo-Ping Ger, Chih-Wen Shu

**Affiliations:** 1 Department of Biomedical Science and Environmental Biology, Kaohsiung Medical University, Kaohsiung 80708, Taiwan; pfliu908203@gmail.com; 2 Institute of Biomedical Sciences, National Sun Yat-sen University, Kaohsiung 80424, Taiwan; lpger0329@gmail.com; 3 Division of Oral & Maxillary Surgery, Department of Stomatology, Kaohsiung Veterans General Hospital, Kaohsiung 81362, Taiwan; 261463@gmail.com (H.-C.C.); angioadsc@gmail.com (C.-H.L.); 4 Department of Internal Medicine, Kaohsiung Veterans General Hospital, Kaohsiung 81362, Taiwan; rcheng@ms2.hinet.net (J.-S.C.); wltsai@vghks.gov.tw (W.-L.T.); 5 School of Medicine, National Yang-Ming University, Taipei 11221, Taiwan; 6 Department of Pathology and Laboratory Medicine, Kaohsiung Veterans General Hospital, Kaohsiung 81362, Taiwan; hplee0627@vghks.gov.tw; 7 School of Medicine for International Students, I-Shou University, Kaohsiung 82445, Taiwan; wansuchi@isu.edu.tw (S.-C.W.); pengweihao@isu.edu.tw (W.-H.P.); 8 Department of Medical Education and Research, Kaohsiung Veterans General Hospital, Kaohsiung 81362, Taiwan

**Keywords:** ATG4B, phosphorylation, autophagy, subsites, tumorigenesis, prognosis, oral cancer

## Abstract

Oral squamous cell carcinoma (OSCC) is one of the major leading causes of cancer death worldwide due to the limited availability of biomarkers and therapeutic targets. Autophagy related protease 4B (ATG4B) is an essential protease for the autophagy machinery, and ATG4B phosphorylation at Ser383/392 increases its proteolytic activity. ATG4B expression and activation are crucial for cancer cell proliferation and invasion. However, the clinical relevance of ATG4B and phospho-Ser383/392-ATG4B for OSCC remains unknown, particularly in buccal mucosal SCC (BMSCC) and tongue SCC (TSCC). With a tissue microarray comprising specimens from 428 OSCC patients, including 179 BMSCC and 249 TSCC patients, we found that the protein levels of ATG4B and phospho-Ser383/392-ATG4B were elevated in the tumor tissues of BMSCC and TSCC compared with those in adjacent normal tissues. High protein levels of ATG4B were significantly associated with worse disease-specific survival (DSS) in OSCC patients, particularly in patients with tumors at advanced stages. In contrast, phospho-Ser383/392-ATG4B expression was correlated with poor disease-free survival (DFS) in TSCC patients. Moreover, ATG4B protein expression was positively correlated with phospho-Ser383/392-ATG4B expression in both BMSCC and TSCC. However, high coexpression levels of ATG4B and phospho-Ser383/392-ATG4B were associated with poor DFS only in TSCC patients, whereas they had no significant association with DSS in BMSCC and TSCC patients. In addition, silencing ATG4B with an antisense oligonucleotide (ASO) or small interfering RNA (siRNA) diminished cell proliferation of TW2.6 and SAS oral cancer cells. Further, knockdown of ATG4B reduced cell migration and invasion of oral cancer cells. Taken together, these findings suggest that ATG4B might be a biomarker for diagnosis/prognosis of OSCC and a potential therapeutic target for OSCC patients.

## 1. Introduction

Oral cancer is the most common type of malignant head and neck cancer. Approximately 90% of oral cancer patients are diagnosed with oral squamous cell carcinoma (OSCC), with approximately 300,000 new cases worldwide every year [1]. There are many types of OSCC, depending on the anatomic location. The tongue is the most common location of all OSCC in the United States and Europe [2]. The buccal mucosa is another common location in OSCC in South Asia. The common risk factors include excessive alcohol consumption, smoking, and human papillomavirus (HPV) [3], whereas betel chewing seems to be the unique cause in Taiwan. Though traditional radiation and chemotherapy provide benefits for OSCC patients, the five-year morbidity rate is still high in patients after diagnosis, particularly for advanced-stage patients (>50%) [1,4], indicating that the previously reported OSCC markers are not convincing in the clinic, likely due to (i) the limited case number from which they were derived, (ii) poor validation of the cause of their appearance or upregulation or of their effect, and (iii) different risk factors in different countries, particularly, betel chewing in Taiwan, which might be associated with different markers. These reports suggest that a more precise therapy for OSCC is needed.

Autophagy is a self-clearance pathway that eliminates abnormal organelles and proteins from cells and is essential for normal physiology. Dysfunction of autophagy is highly associated with many diseases, particularly cancer [5,6]. Autophagy is like a double-edged sword in cells. Growing evidence shows that it serves as a tumor promoter to allow cancer cells to survive in nutrient-deprived or stressed conditions, such as in the central part of tumors and during angiogenesis and induction of cancer stemness [5,7,8]. There are more than 38 autophagy related (ATG) proteins required for autophagy progression in cells. ATG4 is a cysteine protease involved in the formation of lipid-bound MAP1LC3 (MAP1LC3-II) and recycling of MAP3LC3-II from appropriated autophagosomes [9]. ATG4B is the most active member of the ATG4 family (ATG4A, ATG4B, ATG4C, and ATG4D) in mammalian cells [10]. The oncogenic role of ATG4B in cancers has been reported previously in cell culture, tumor xenograft models, and even in clinical settings [11,12,13,14]. ATG4B overexpression is associated with cancer development in patients with colorectal cancer and chronic myeloid lymphoma (CML) [11,15]. Silencing ATG4B decreases tumor growth of colorectal cancer and cancer stemness of cluster of differentiation 34(CD34^+^) CML [11,15]. Moreover, knockdown of ATG4B also increases sensitivity to trastuzumab in HER2-positive breast cancer cells [16]. Overexpression of catalytic mutant ATG4B^C74A^ arrests cell growth of glioma and hepatocellular carcinoma cells, indicating that proteolysis of ATG4B is essential for tumor growth [12,17]. Moreover, mutation of ATG4B at Ser383/392 leads to the loss of ATG4B proteolytic activity in cells [18]. These results suggest that ATG4B might be crucial in tumor progression. However, little is known about the clinical relevance of ATG4B, particularly of phosphorylated ATG4B, in patients with OSCC.

In this study, we compared the ATG4B and phospho-Ser383/392-ATG4B protein levels between tumor tissues and adjacent normal tissues in buccal mucosal SCC (BMSCC) and tongue SCC (TSCC). We found that both ATG4B and phospho-Ser383/392-ATG4B were elevated in tumor tissues of OSCC and associated with poor prognosis in certain stages of OSCC. Furthermore, a correlation between ATG4B and phospho-Ser383/392-ATG4B was found in OSCC patients. To determine the potential function of ATG4B in BMSCC and TSCC cell lines, ATG4B was silenced with antisense oligonucleotide (ASO) or small interfering RNA (siRNA), and the effects on cell proliferation, migration, and invasion in oral cancer cells were measured. Our results support the oncogenic role of ATG4B in OSCC and suggest that ATG4B and phospho-Ser383/392-ATG4B could serve as biomarkers or therapeutic targets for OSCC.

## 2. Experimental Procedure

### 2.1. Tissue Specimens and Tissue Microarray (TMA) Construction

In total, 428 tissues obtained from primary BMSCC (*n* = 179) and TSCC (*n* = 249) between 1993 and 2006 were embedded in paraffin to construct a TMA, as described previously [19] Each patient tissue contained 1 core of adjacent normal tissue and 2 cores of tumor tissue in the TMA block. The blocks were cut in 4 μm paraffin sections. Clinical information for each patient was collected, including sex, age, and tumor cell differentiation, pathological stage, TNM classification, subsites, and recurrence time, which were also internally collected at Kaohsiung Veterans General Hospital (KVGH). Histologic grades G1, G2–3, and G4 are considered to correspond to well, moderate, and poor cell differentiation, respectively. TNM stages were classified by the guideline of the 8th edition of the American Joint Committee on Cancer (AJCC) Cancer Staging Manual [20]. The Institutional Review Board approved this study, which complies with the Declaration of Helsinki (institutional review board (IRB) number: VGHKS 18-CT1-13).

### 2.2. Immunohistochemistry (IHC)

The TMA blocks were employed for immunohistochemistry staining as previously reported [6]. The TMA blocks were immersed in Tris-Ethylenediamine tetraacetic acid (EDTA) buffer (10 mM, pH 9.0) and boiled in a pressure boiler at 125 °C for 10 min for antigen retrieval of ATG4B and phospho-Ser383/392-ATG4B. Endogenous peroxidases in the TMA slides were blocked with 3% hydrogen peroxide in methanol, and the unspecific binding was prevented by 3% bovine serum albumin (BSA). The slides were then incubated with antibodies against ATG4B (dilution 1:100; A2981, Sigma-Aldrich, St. Louis, MO, USA) or phospho-Ser383/392-ATG4B (dilution 1:100; homemade from a phosphor-peptide immunized rabbit) in a cold room overnight. The specificity of the antibody against phospho-S383/392 ATG4B was validated by a dot blot assay using various concentration of phospho-S383 and phospho-S392 peptide, respectively (Figure S1). The colored immunohistochemistry (IHC) stains for each protein were developed at room temperature and counterstained with hematoxylin.

### 2.3. Immunohistochemistry Analysis and Score

**Figure 1:**
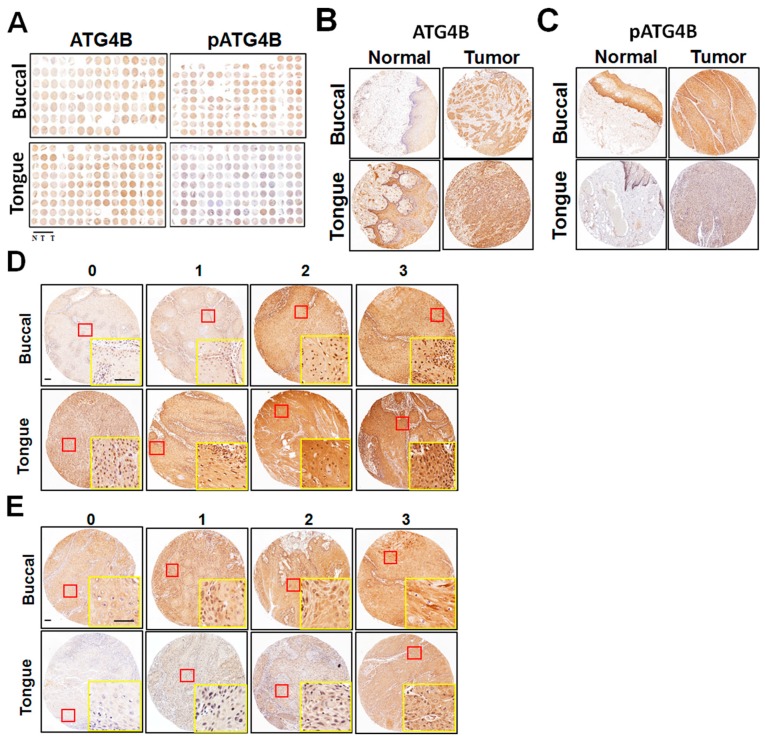
Protein levels of ATG4B and phospho-Ser383/392-ATG4B in oral squamous cell carcinoma (OSCC). (**A**) Tissue microarrays consisting of tissues from 127 buccal mucosal SCC (BMSCC) patients and from 201 tongue SCC (TSCC) patients. Each sample for each patient included one portion of adjacent normal tissue (N) and two portions of tumor tissues (T). Tissue microarrays were stained via immunohistochemistry using antibodies against ATG4B or phospho-Ser383/392-ATG4B (pATG4B). Representative images are shown. (**B**) Representative immunohistochemistry staining of ATG4B or (**C**) phospho-Ser383/392-ATG4B (pATG4B) for paired tumor and adjacent normal tissues from BMSCC and TSCC. (**D**) The staining intensity for ATG4B or (**E**) phospho-Ser383/392-ATG4B (pATG4B) was categorized into four different levels, as the standard slides show: 0 = negative staining; 1 = weak; 2 = moderate; 3 = strong. Yellow rectangle is zoom in from red rectangle. Scale bar for (D) and (E): 100 μm.

All slides were scored twice by a cancer pathologist. Next, 5–20% of the cores were randomly selected for re-evaluation by another senior oral cancer pathologist. If disagreement occurred (intensity score discrepancy >1 or percentage level >20%) between two pathologists, the slides were re-checked until all discrepancies were resolved. The scores for protein levels were categorized into four groups according to the staining intensity (0, no signal; 1, mild; 2, moderate; and 3, strong) and percentage of positive staining (0–100%). The standard intensity score for cytosolic ATG4B and phospho-Ser383/392-ATG4B in OSCC is shown in Figure 1 (0, no expression; 1, weak expression; 2, moderate expression; and 3, strong expression). The final score of each tissue was calculated as intensity multiplied by (percentage ×100), ranging from 0 to 300. For survival analysis, the protein levels were categorized into low and high, using the cutoff based on the receiver operating characteristic (ROC) curve. The cutoff values were individually determined for ATG4B and phospho-Ser383/392-ATG4B in OSCC, BMSCC, and TSCC.

### 2.4. Cell Culture and Transient Transfection

The BMSCC cell line TW2.6 and the TSCC cell line SAS were cultured in plastic dishes (Corning, Inc., Corning, NY, USA) containing DMEM/F12 (Gibco, Life Technologies, CA, USA) with 10% fetal bovine serum (FBS), 100 μg/mL of streptomycin, 100 U/mL of penicillin, and 1% L-glutamine at 37 °C. The cells were reversely transfected with nontargeting antisense oligonucleotide (ASO-Ctrl) or ASO against ATG4B (522147-1, EXIQON, Vedbaek, Denmark) using RNAiMAX (13778150; Life Technologies, Carlsbad, CA, USA) to knock down ATG4B in OSCC cells. Alternatively, the cells were transfected with scrambled siRNA (12935-112, Life Technologies, Carlsbad, CA, USA) or siRNA against ATG4B (20218, s23245, s23246, Life Technologies, Carlsbad, CA, USA) in the presence of RNAiMAX for 72 h. The knockdown efficiency was determined with quantitative PCR and immunoblotting using the ATG4B antibody as previously reported [12,13]. Briefly, 1 × 10^6^ cells were harvested to analyze mRNA and protein levels. Then, 1 μg of RNA was reverse-transcribed for cDNA synthesis, and ATG4B mRNA level was quantified relative to GAPDH with real-time PCR (StepOnePlusTM system, Applied Biosystems, Waltham, MA, USA). For immunoblotting, the cells were lyzed with RIPA buffer in the presence of a protease inhibitor cocktail (Roche, 11873580001)). Then, 50 μg of total proteins was boiled with 2 × SDS sample buffer (100 mM Tris-Cl (pH 6.8), 4% (*w/v*) SDS, 0.2% (*w/v*) bromophenol blue) and used for immunoblotting with primary antibodies against ATG4B (A2981) and ACTB (β-actin, A5441) (Sigma-Aldrich, St. Louis, MI, USA). The protein expression levels were detected with HRP-labeled secondary antibodies (Santa Cruz Biotechnology, Dallas, TX, USA, sc-2004 or sc-2005) and the Enhanced chemiluminescence (ECL, Thermo Fisher Scientific, Carlsbad, CA, USA) reagent. The luminescent signal was analyzed to determine the protein expression levels using the ChemiDoc XRS Imaging System (Bio-Rad, Hercules, CA, USA).

### 2.5. Analysis of Cell Viability, Migration, and Invasion

Cell viability was determined by the CellTiter-Glo luminescent cell viability assay kit (Promega, Madison, WI, USA) as described previously [21]. Briefly, 1 × 10^5^ cells/mL were seeded in 96-well plates with ASO or siRNA in a CO_2_ incubator for 48–72 h. The cells were then lysed and measured with 100 μL of CellTiter-Glo reagent for detection of cellular ATP levels, reflecting cell proliferation. The luminescence signal was read by a Fluoroskan Ascent FL reader (Thermo Fisher Scientific, Carlsbad, CA, USA). For the wound healing assay, the cells were transfected with siRNA for 48 h in a T25 culture flask. The cells (1.5 × 10^5^/140 μL) were then plated in culture inserts (IBIDI, Inc., Planegg, Germany) for 16 h [22]. Afterward, the inserts were removed to allow cells to migrate for 4 h. The distance covered by moving cells from time 0 to 4 h was counted as migration distance. We used 8 μm pore inserts (Greiner Bio-One, Stroud, UK) to determine the invasion capability of cancer cells as described before [14]. Briefly, Transwell chambers were coated with 0.5% Matrigel, and silenced cells were seeded in them in 300 μL of Dulbecco’s Modified Eagle’s Medium (DMEM) containing 1% Fetal Bovine Serum (FBS). After invasion, the invaded cells that were attached to the bottom of the chamber were stained with 0.1% crystal violet and observed under a microscope at a magnification of 200×. Images were quantified by ImageJ software and analyzed by Prism 5 software (GraphPad, San Diego, CA, USA).

### 2.6. Statistical Analysis

The protein levels of ATG4B and phospho-Ser383/392-ATG4B in tumor tissues and adjacent normal tissues were analyzed by the Wilcoxon signed-rank test. The correlation between the protein levels of ATG4B or phospho-Ser383/392-ATG4B and clinicopathologic parameters was analyzed by the Student’s *t* test, the Mann-Whitney U test, the Kruskal-Wallis one-way ANOVA test, and the one-way ANOVA test. The correlation between ATG4B and phospho-Ser383/392-ATG4B levels was estimated by Spearman’s rank correlation coefficient. Cumulative survival curves were evaluated by the Kaplan-Meier method and investigated for significance by the log-rank test. The association of protein levels with disease-specific survival (DSS) or disease-free survival (DFS) of patients adjusted for cell differentiation (moderate + poor vs. well) and AJCC pathological stage (stage III + IV vs. stage I + II) was investigated by a multivariate Cox regression model. A two-sided value of *p* < 0.05 was considered statistically significant. For cell culture experiments, the results were obtained from three independent experiments (*n* = 3 per each experiment), and the significance was calculated by a non-parametric 2-tailed Student’s *t*-test.

## 3. Results

### 3.1. Association of the ATG4B and Phospho-Ser383/392-ATG4B Protein Levels with Tumorigenesis and Clinicopathological Outcomes

The expression levels of ATG4B and the potential active form of phospho-Ser383/392-ATG4B were initially verified by IHC staining of a TMA containing samples from OSCC patients and including tissues from two major anatomic locations, which corresponded to BMSCC and TSCC. Representative staining slides for ATG4B and phospho-Ser383/392-ATG4B are shown (Figure 1A). One core of adjacent normal tissue and two cores of tumor tissue were obtained from each patient. The stained paired adjacent normal tissues and tumor tissues of BMSCC or TSCC were observed under a microscope (Figure 1B,C). The standard intensity score of ATG4B (Figure 1D) or phospho-Ser383/392-ATG4B (Figure 1E) in BMSCC and TSCC was recorded as described in the Methods section. The total score for each tissue was defined as the staining intensity multiplied by the staining percentage. The levels of the examined proteins were higher in OSCC tumor tissues than in adjacent normal tissues. ATG4B levels were 165.75 ± 54.67 vs. 145.15 ± 70.9, *p* < 0.001, and phospho-Ser383/392-ATG4B levels were 168.65 ± 74.32 vs. 111.66 ± 84.17, *p* < 0.001, (Table 1). Phospho-Ser383/392-ATG4B level was predominantly increased in tumor tissues compared with adjacent normal tissues in BMSCC (230.85 ± 53.91 vs. 175.87 ± 73.63, *p* < 0.001) and TSCC (129.35 ± 56.48 vs. 71.09 ± 62.42, *p* < 0.001), suggesting that ATG4B expression and activity might be involved in the tumorigenesis of OSCC.

Moreover, we analyzed the correlation of ATG4B and phospho-Ser383/392-ATG4B protein levels with clinicopathological outcomes at two subsites of OSCC. ATG4B expression was positively associated with lymph node invasion in OSCC (*p* = 0.022), mainly in TSCC (*p* = 0.049, Table 2). In contrast, phospho-Ser383/392-ATG4B was associated with many clinicopathological outcomes in patients with OSCC, including sex (*p* = 0.006), cell differentiation (*p* < 0.001), pathological stage (*p* = 0.043), and T classification (*p* = 0.044, Table 3). However, it was not associated with any clinicopathological outcomes in BMSCC and TSCC.

### 3.2. Protein Levels of ATG4B and Phospho-Ser383/392-ATG4B and DSS of OSCC Patients

**Figure 2:**
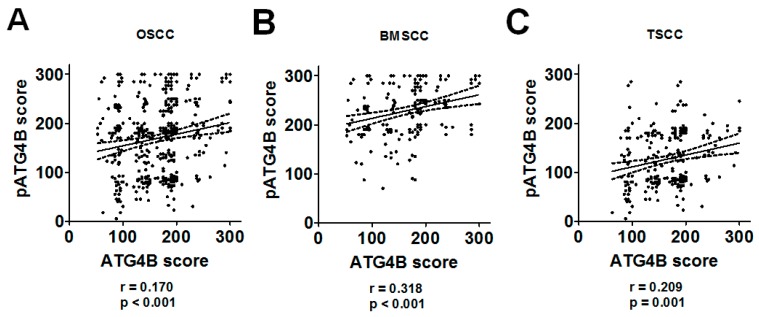
Correlation between ATG4B and phospho-Ser383/392-ATG4B protein levels in patients with BMSCC or TSCC. (**A**) The correlation between the protein levels of ATG4B and phospho-Ser383/392-ATG4B (pATG4B) in all OSCC patients was determined by Pearson analysis (*r* = 0.170, *p* < 0.001). (**B**) The tumor tissues of OSCC patients were further divided into BMSCC (*r* = 0.318, *p* < 0.001) and (**C**) TSCC (*r* = 0.209, *p* = 0.001), and the correlation with the corresponding subsites of OSCC was inspected.

**Figure 3:**
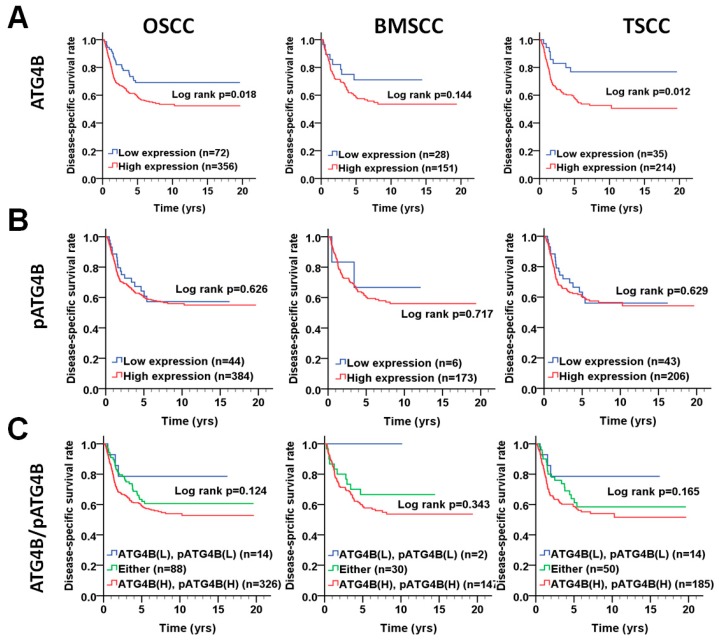
Disease-specific survival (DSS) survival curves in relation to ATG4B and phospho-Ser383/392-ATG4B protein levels in patients with BMSCC or TSCC. (**A**) DSS survival curves in relation to ATG4B or (**B**) phospho-Ser383/392-ATG4B (pATG4B) are shown for OSCC (left panel), BMSCC (middle), and TSCC (right panel) patients. (**C**) DSS survival curves in relation to the coexpression of ATG4B and phospho-Ser383/392-ATG4B are shown for OSCC, BMSCC, and TSCC patients. The cutoff values for high (H) or low (L) protein levels of ATG4B and phospho-Ser383/392-ATG4B in tumor tissues were based on the receiver operating characteristic (ROC) curve.

We further investigated whether the protein levels of ATG4B are correlated with those of phospho-Ser383/392-ATG4B in tumor tissues of OSCC (Figure 2A). Pearson’s correlation analysis results indicated that ATG4B expression had a moderate positive correlation with phospho-Ser383/392-ATG4B expression in the tumor tissues of BMSCC (Figure 2B), while it showed a low positive correlation with phospho-Ser383/392-ATG4B expression in TSCC (Figure 2C). Moreover, to determine whether ATG4B and phospho-Ser383/392-ATG4B could be used as biomarkers for prognosis for two subsites of OSCC, the relationship of ATG4B and phospho-Ser383/392-ATG4B with DSS was initially examined by Kaplan-Meier curve analysis (Figure 3). The results showed that higher ATG4B expression was associated with worse DSS in OSCC (Figure 3A, *p* = 0.018), mostly in TSCC (*p* = 0.012, Figure 3A). Phospho-Ser383/392-ATG4B (Figure 3B) or coexpression with ATG4B (Figure 3C) was not associated with poor DSS. We further stratified ATG4B expression with DSS according to different clinicopathological features and found that high expression of ATG4B was associated with poor DSS in males (adjusted hazard ratio(AHR): 1.74, *p* = 0.016, Table 4), elderly patients (>50) (AHR: 2.15, *p* = 0.013), patients with poorly differentiated tumors (AHR: 2.05, *p* = 0.004), advanced-stage patients, such as those with AJCC pathological stages III + IV tumors (AHR: 2.34, *p* = 0.008), and T classification (AHR: 3.10, *p* = 0.010). Interestingly, higher ATG4B expression was associated with a worse DSS rate in OSCC patients without radiation therapy (AHR: 2.55, *p* = 0.004). Through multiple Cox regression analyses with adjustments for cell differentiation (moderate + poor vs. well) and AJCC pathological stage (stage III + IV vs. stage I + II), patients with higher ATG4B expression had shorter DSS, for both BMSCC (AHR: 2.70, 95% confidence interval (CI): 1.28-5.68, *p* = 0.009) and TSCC (AHR: 2.19, 95% CI: 1.06–4.52, *p* = 0.034, Table 5). However, neither the protein level of phospho-Ser383/392-ATG4B nor its coexpression with ATG4B had significant effects on DSS in either BMSCC or TSCC (Figure 3, Table 5 and Table A1), implying that ATG4B expression was more important than its activity for DSS of OSCC patients.

### 3.3. Association of the ATG4B and Phospho-Ser383/392-ATG4B Protein Levels with Disease-Free Survival in OSCC Patients

**Figure 4:**
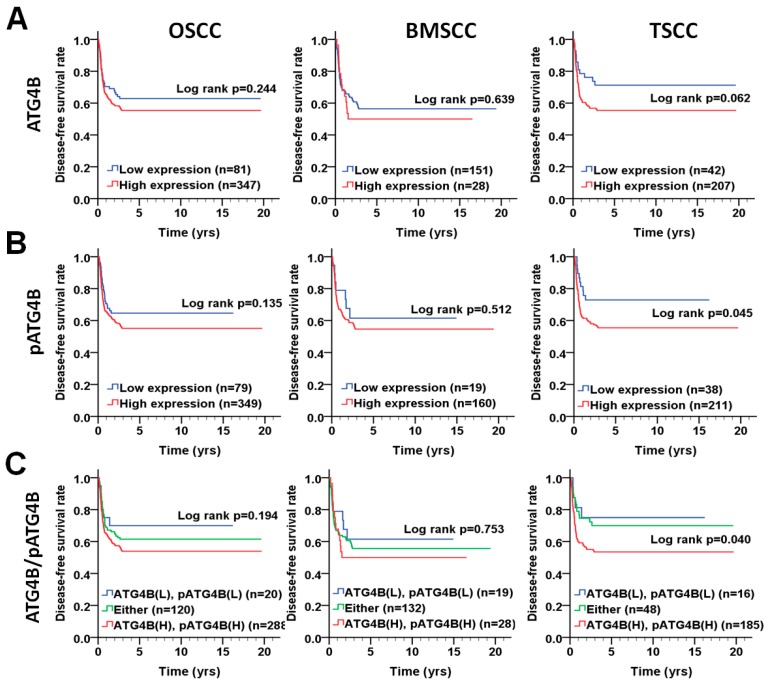
DFS survival curves for ATG4B and phospho-Ser383/392-ATG4B expression in patients with BMSCC or TSCC. (**A**) DFS survival curves in relation to ATG4B or (**B**) phospho-Ser383/392-ATG4B (pATG4B) expression are shown for OSCC (left panel), BMSCC (middle), and TSCC (right panel) patients. (**C**) DFS survival curves for the coexpression of ATG4B and phospho-Ser383/392-ATG4B are shown for OSCC, BMSCC, and TSCC patients. The cutoff values for high or low protein levels of ATG4B and phospho-Ser383/392-ATG4B in tumor tissues were based on the ROC curve.

To determine whether ATG4B and phospho-Ser383/392-ATG4B are correlated with relapse in OSCC, we further analyzed the association of ATG4B and phospho-Ser383/392-ATG4B protein levels with disease-free survival (DFS) using the Kaplan–Meier curve (Figure 4). The results showed that high protein levels of phospho-Ser383/392-ATG4B alone (*p* = 0.045, Figure 4B) or coexpression (*p* = 0.040, Figure 4C) with ATG4B were notably associated with a shorter DFS in TSCC patients. However, ATG4B expression alone or coexpression with phospho-Ser383/392-ATG4B was not correlated with DFS in either BMSCC or TSCC patients (Figure 4A,C and Table A2). Stratification analysis indicated that high phospho-Ser383/392-ATG4B expression significantly worsened DFS in OSCC patients with AJCC pathological stages III + IV (AHR: 2.59, *p* = 0.040, Table 6). These results suggested that the phosphorylation form of ATG4B might be crucial for tumor relapse in patients with TSCC but not in those with BMSCC. Similarly, Cox regression analysis with adjustments for cell differentiation and AJCC pathological stage showed that an increased protein level of phospho-Ser383/392-ATG4B had a higher hazard ratio in patients with TSCC (AHR: 1.95, *p* = 0.045, Table 7).

### 3.4. Function of ATG4B in Cell Proliferation and Invasion of OSCC Cells

**Figure 5:**
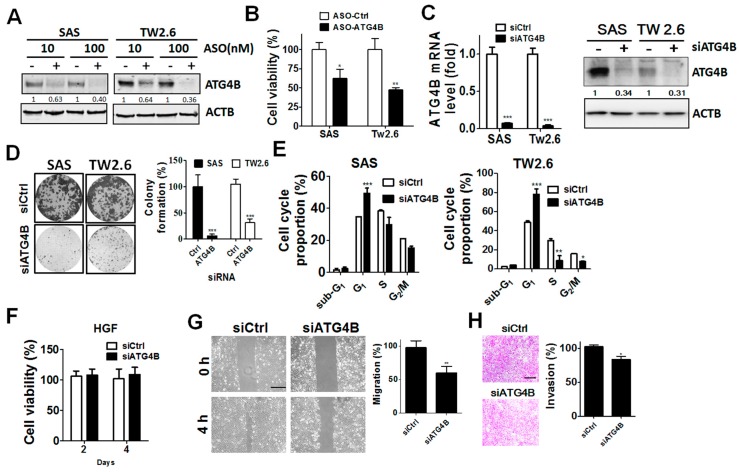
Effects of silencing ATG4B on cell proliferation and invasion in oral cancer cells. (**A**) The TSCC cell line SAS and the BMSCC cell line TW2.6 were transfected with scrambled (−) antisense oligonucleotide (ASO) or ASO against ATG4B (+) for 72 h, and silencing was verified by immunoblotting. (**B**) The cell proliferation of 10 nM ASO-silenced cells was determined by analyzing cellular ATP levels using the CellTiter Glo assay. The quantitative results are expressed as the mean ± SD from three independent experiments. * *p* < 0.05, ** *p* < 0.01 vs. non-targeting control ASO (ASO-Ctrl). (**C**) OSCC cells were transfected with scramble siRNA (−, 5 nM) or siRNA against ATG4B (+, 5 nM) for 48 h. Knockdown efficiency was determined by quantitative PCR (left panel) or immunoblotting (right panel). (**D**) The transfected cells were grown until colony formation. The colonies are shown in the left panel, and their quantification is shown in the right panel. The quantitative results are expressed as the mean ± SD from three independent experiments. *** *p* < 0.001 vs. non-targeting control siRNA (siCtrl). (**E**) Silenced cells were stained with propidium iodide for cell cycle analysis using flow cytometry. The cell cycle proportion was quantitated with Prism 5.0. (**F**) Human normal gingival fibroblast (HGF) cells were transfected with 5 nM scramble siRNA or siRNA against ATG4B and analyzed by a cell viability assay. (**G**) SAS oral cancer cells were silenced for 48 h and then plated in culture inserts for migration assays. ** *p* < 0.01 vs. non-targeting control siRNA (siCtrl). (**H**) The transfected cells were cultured in Matrigel-coated Transwell filters for 8 h to assess cell invasion. The quantitative results are expressed as the mean ± SD from three independent experiments. * *p* < 0.05 vs. non-targeting control siRNA (siCtrl). The effects of silencing ATG4B on cell viability, migration, and invasion of oral cancer cells were evaluated as percentage using cells with scramble ASO (100%) or siRNA. Scale bar for (G) and (H): 100 μm

Given the clinical results mentioned above, ATG4B and phospho-Ser383/392-ATG4B protein levels were elevated in tumor tissues compared with adjacent normal tissues in both BMSCC and TSCC. To determine the potential function of ATG4B in cell proliferation, ATG4B expression was silenced in TW2.6 BMSCC and SAS TSCC cells with scrambled ASO (control) or ASO against ATG4B (Figure 5A,B and Figure S2). Both TW2.6 and SAS ATG4B-knockdown cells had significantly reduced cell viability. The knockdown efficiency of siRNA against ATG4B at mRNA and protein levels in oral cancer cells was confirmed by quantitative PCR and immunoblotting (Figure 5C). Silencing ATG4B significantly inhibited cell growth and arrested cell cycle progression in G1 phase in both TW2.6 BMSCC cells and SAS TSCC cells (Figure 5D,E and Figure S2). Interestingly, knockdown of ATG4B did not alter cell proliferation in human normal gingival cells (HGF) (Figure 5F). Moreover, ATG4B expression was positively correlated with lymph node invasion of TSCC, as shown in Table 2, implying that ATG4B may be contribute to the invasive characteristics of cancer cells. We further inspected the involvement of ATG4B in the migration and invasion of SAS cells (Figure 5G). Knockdown of ATG4B significantly reduced the migration of SAS cells (Figure 5G), while their invasion ability was modestly diminished (Figure 5H). These results imply that ATG4B might modulate cell growth and invasion ability of TSCC cells.

## 4. Discussion

ATG4B promotes cell cycle progression from G_1_ phase to S phase transition, drug resistance, and stemness in cancer cells [12,13,15]. The proteolytic activity of ATG4B is involved in cancer cell malignancy [12]. Phosphorylation of ATG4B at Ser383 and Ser392 is important for full activation of its proteolytic activity [18]. However, little is known about the clinical relevance of ATG4B and phospho-Ser383/392-ATG4B in patients with OSCC, particularly for patients with tumors in the major anatomic locations, i.e., BMSCC and TSCC. Here, we reported the following findings: First, the protein levels of ATG4B and phospho-Ser383/392-ATG4B were significantly elevated in tumor tissues compared with adjacent normal tissues in both BMSCC and TSCC. Second, ATG4B was associated with lymph node invasion and unfavorable DSS for BMSCC and TSCC patients, while phospho-Ser383/392-ATG4B was associated with poor DFS. Third, ATG4B expression had a significantly positive correlation with phospho-Ser383/392-ATG4B expression in BMSCC and TSCC. Kaplan–Meier analysis indicated that higher coexpression of ATG4B and phospho-Ser383/392-ATG4B was correlated with shorter DFS. Fourth, silencing ATG4B with either ASO or siRNA attenuated cell proliferation, migration and invasion of OSCC cells. To the best of our knowledge, we are the first group to report the relationship of ATG4B and phospho-Ser383/392-ATG4B expression with clinicopathological outcomes in BMSCC and TSCC patients from one of the largest OSCC cohorts worldwide.

In addition to oxidative regulation of ATG4B activity, numerous posttranslational modifications of ATG4B have been reported previously, mainly phosphorylation [23,24,25]. The Unc-51-like autophagy activating kinase 1 (ULK1) complex plays a crucial role in phagophore initiation during autophagy. A recent report shows that it interacts with ATG4B and phosphorylates Ser316 of ATG4B to inhibit proteolytic activity at sites of autophagosome formation, whereas PP2A can reversely reactivate ATG4B activity in mammalian cells [23]. Screening with a small-molecule inhibitor library identified AKT2 as a potential kinase for ATG4B activation. A recent study indicated that AKT1 phosphorylates ATG4B at Ser 34 and that phosphorylation has little effect on autophagic flux [25]. Phosphorylation of ATG4B at Ser383/393 is required for full proteolytic activity [18]. Furthermore, MST4 can phosphorylate ATG4B at Ser383 to induce autophagy and resistance to radiotherapy in glioblastoma cells [26]. However, none of these phosphorylation sites has been reported to be relevant in clinical settings. Our current results showed that phospho-Ser383/392-ATG4B expression was elevated in tumor tissues and was associated with DFS in patients with TSCC. Together with previous reports, this raises the possibility that the upstream kinase for ATG4B Ser383 phosphorylation might play a vital role in the tumorigenesis and relapse of TSCC, which requires further study.

ATG4B is the major autophagy-related protease required for activation of membrane-bound MAP1LC3-II, which is crucial for autophagosome elongation in mammalian cells. Our recent study showed that high protein levels of MAP1LC3-II puncta are linked to unfavorable DSS in TSCC but not in BMSCC [6]. In line with our present study, ATG4B expression was associated with poor DSS in TSCC patients, implying that ATG4B expression might be positively correlated with MAP1LC3-II puncta for autophagy induction and cause poor outcome in TSCC patients. In contrast, ATG4B is also essential for the removal of MAP1LC3-II from autophagosomes to recycle MAP31LC3 during autophagy [27]. ATG4B could be transiently inactivated by ROS to promote autophagy in cells during starvation [28]. Additionally, phosphorylation of ATG4B at Ser383/392, generating an active form of ATG4B, was not correlated with DSS in patients with TSCC, suggesting that ATG4B activity in autophagy might be in a spatiotemporal limiting step or that other posttranslational modifications of ATG4B interfere with activation. Thus, the protease activity of ATG4B in formalin-fixed tissues might not completely reflect the autophagic flux in tumor tissues. These results suggest that elucidating the role of ATG4B-modulated autophagy in tumor tissues may require more studies. Regardless of autophagy activity, our current results indicate that ATG4B was associated with poor DSS and that phospho-Ser383/392 was associated with DFS in patients with TSCC. On the other hand, silencing ATG4B or overexpressing an ATG4B catalytic mutant (ATG4B^C74A^) increased adenosine monophosphate (AMP)-activated protein kinase (AMPK) phosphorylation, inactivated mammalian target of rapamycin complex (MTORC), and induced p27^kip1^ accumulation leading to cell cycle arrest in the G1 phase in colorectal cancer cells [12]. Furthermore, autophagy promotes epithelial-mesenchymal transition (EMT) via modulation of several crucial factors of EMT, such as vimentin, TWIST, and E-cadherin [29]. Thus, the detailed mechanisms of action of ATG4B affecting cell proliferation and invasion in oral cancer cells, particularly in clinical setting, requires further study.

In the survival analysis, the number of patients in the low-expression group of ATG4B or phospho-Ser383/392-ATG4B was much lower than the number in the high-expression group, which may cause biases. The protein levels of ATG4B and phospho-Ser383/392-ATG4B were categorized into low and high levels according to the ROC curve, which is an unbiased way to separate patients into two groups. Our study showed that high levels of ATG4B were correlated with poor DSS, while phospho-Ser383/392-ATG4B expression was associated with DFS in TSCC patients.

## 5. Conclusions

Our results indicate ATG4B as a potential biomarker for the diagnosis or prognosis of TSCC. On the other hand, silencing ATG4B significantly attenuated growth, migration, and invasion of oral cancer cells, suggesting ATG4B as a potential target for oral cancer therapy. In addition, various high-throughput screening methods have identified several ATG4B inhibitors, including the clinical drug tioconazole, the *Xanthium strumarium* fruit extract, and the small molecule S130 [9,13,14]. These ATG4B inhibitors can suppress colorectal cancer cell growth and synergize the killing effect of cancer cells with autophagy-induction. These results imply that ATG4B inhibition might be able to block proliferation and sensitize oral cancer cells to chemotherapeutic drugs or radiation therapy, but this hypothesis would need to be evaluated with further study.

## Figures and Tables

**Table 1:** Comparison of ATG4B and pATG4B expression between tumor tissues and corresponding tumor-adjacent normal tissues at two subsites of OSCC.

Variables	No.	Tumor-Adjacent Normal		Tumor		Z	*p*-Value^*^
		Mean ± SD	Median	Mean ± SD	Median		
OSCC							
ATG4B	328	145.15 ± 70.90	160.00	165.75 ± 54.67	177.50	4.578	<0.001
pATG4B	328	111.66 ± 84.17	85.00	168.65 ± 74.32	176.25	11.302	<0.001
BMSCC							
ATG4B	127	146.81 ± 73.35	160.00	166.02 ± 62.44	180.00	2.665	0.008
pATG4B	127	175.87 ± 73.63	180.00	230.85 ± 53.91	237.50	6.114	<0.001
TSCC							
ATG4B	201	144.10 ± 69.48	160.00	165.58 ± 49.29	175.00	3.781	<0.001
pATG4B	201	71.09 ± 62.42	70.00	129.35 ± 56.48	122.50	9.291	<0.001

Abbreviations: SD, standard deviation. ^*^ *p-*values were estimated by Wilcoxon signed-rank test. OSCC, oral squamous cell carcinoma; BMSCC, buccal mucosal squamous cell carcinoma; TSCC, tongue squamous cell carcinoma

**Table 2:** Expression of ATG4B and clinicopathologic outcomes in patients with OSCC.

Variable	%	OSCC (*n* = 428)			%	BMSCC (*n* = 179)			%	TSCC (*n* = 249)		
		Mean ± SD	Median	*p* Value		Mean ± SD	Median	*p* Value		Mean ± SD	Median	*p* Value
Sex												
Female	7.9	170.76 ± 49.89	190.00	0.642 *	2.2	179.38 ± 32.04	192.50	0.662 *	12.0	169.61 ± 52.10	190.00	0.744 *
Male	92.1	166.27 ± 54.25	180.00		97.8	165.99 ± 60.84	180.00		88.0	166.50 ± 48.49	175.00	
Age, y												
≤40	15.4	165.61 ± 51.38	175.00	0.166 ^†^	13.4	183.44 ± 57.10	180.00	0.105 ^†^	16.9	155.42 ± 45.43	152.50	0.212 ^†^
41–50	31.8	165.19 ± 56.47	170.00		30.7	158.09 ± 64.01	150.00		32.5	170.01 ± 50.57	180.00	
51–60	29.0	160.64 ± 52.92	180.00		30.2	156.76 ± 56.35	180.00		28.1	163.63 ± 50.33	177.50	
>60	23.8	176.50 ± 52.48	185.00		25.7	178.32 ± 59.83	185.00		22.5	175.00 ± 46.08	185.00	
Subsites												
Buccal	41.8	166.28 ± 60.33	180.00	0.911 *	100.0	166.28 ± 60.33	180.00					
Tongue	58.2	166.88 ± 48.84	180.00						100.0	166.88 ± 48.84	180.00	
Cell differentiation												
Well	17.8	164.97 ± 61.05	177.50	0.558 ^†^	27.4	165.71 ± 63.12	180.00	0.988 ^†^	10.8	163.61 ± 58.24	175.00	0.287 ^†^
Moderate	76.2	166.15 ± 51.71	180.00		67.6	166.69 ± 60.39	180.00		82.3	165.82 ± 45.98	180.00	
Poor	6.1	177.56 ± 59.18	185.00		5.0	163.89 ± 48.67	185.00		6.8	184.80 ± 64.24	195.00	
AJCC pathological stage												
I	31.8	165.66 ± 52.14	180.00	0.803 ^†^	35.2	168.06 ± 54.54	180.00	0.806 ^†^	29.3	163.60 ± 50.26	180.00	0.567 ^†^
II	33.4	164.73 ± 52.08	175.00		26.3	165.64 ± 64.73	180.00		38.6	164.29 ± 45.00	170.00	
III	15.2	172.51 ± 60.25	180.00		10.6	176.32 ± 67.70	180.00		18.5	170.94 ± 57.63	180.00	
IV	19.6	166.88 ± 55.11	180.00		27.9	160.85 ± 61.40	177.50		13.7	175.74 ± 43.67	190.00	
T classification												
T1	33.9	164.98 ± 51.80	180.00	0.389 ^†^	38.0	165.22 ± 54.59	177.50	0.368 ^†^	30.9	164.77 ± 49.58	180.00	0.518 ^†^
T2	43.7	171.19 ± 55.78	180.00		38.0	175.00 ± 66.21	185.00		47.8	169.02 ± 49.00	175.00	
T3	11.2	157.85 ± 46.59	175.00		6.7	154.79 ± 44.19	175.00		14.5	158.87 ± 47.93	167.50	
T4	11.2	162.60 ± 58.99	182.50		17.3	153.95 ± 63.65	170.00		6.8	178.38 ± 47.10	190.00	
N classification												
N0	77.3	163.40 ± 52.99 ^a^	175.00	0.022 ^†^	74.9	163.34 ± 60.07	177.50	0.321 ^†^	79.1	163.44 ± 47.74	175.00	0.049 ^†^
N1	10.7	186.45 ± 60.89 ^a^	185.00		12.3	184.32 ± 66.05	185.00		9.6	188.40 ± 57.12	183.75	
N2	11.9	169.71 ± 49.80	180.00		12.8	166.20 ± 55.66	180.00		11.2	172.59 ± 45.26	187.50	

Abbreviations: AJCC, American Joint Committee on Cancer, T: tumor. ^*^ *p* values were estimated by Student’s *t*-test. ^†^ *p* values were estimated by one-way ANOVA test.

**Table 3:** Expression of pATG4B and clinicopathologic outcomes in patients with OSCC.

Variable	%	OSCC (*n* = 428)			%	BMSCC (*n* = 179)			%	TSCC (*n* = 249)		
		Mean ± SD	Median	*p* Value		Mean ± SD	Median	*p* Value		Mean ± SD	Median	*p* Value
Sex												
Female	7.9	135.83 ± 57.99	127.50	0.006 *	2.2	227.50 ± 46.99	222.50	0.950 *	12.0	123.61 ± 57.99	105.00	0.706 *
Male	92.1	172.83 ± 74.95	180.00		97.8	229.21 ± 54.19	235.00		88.0	127.78 ± 56.49	125.00	
Age, y												
≤40	15.4	159.37 ± 73.07	170.00	0.606 ^†^	13.4	220.00 ± 59.01	235.00	0.850 ^†^	16.9	124.72 ± 55.94	107.92	0.410 ^†^
41–50	31.8	174.34 ± 73.33	182.50		30.7	230.86 ± 52.98	237.50		32.5	135.96 ± 59.08	135.00	
51–60	29.0	169.21 ± 76.90	175.00		30.2	230.60 ± 57.25	236.25		28.1	121.85 ± 52.91	130.00	
>60	23.8	171.61 ± 75.91	180.00		25.7	230.27 ± 49.43	232.50		22.5	123.42 ± 57.80	92.50	
Subsites												
Buccal	41.8	229.18 ± 53.93	235.00	<0.001 *	100.0	229.18 ± 53.93	235.00					
Tongue	58.2	127.28 ± 56.57	122.50						100.0	127.28 ± 56.57	122.50	
Cell differentiation												
Well	17.8	201.41 ± 73.80 ^ab^	195.00	<0.001 ^†^	27.4	235.31 ± 53.89	245.00	0.065 ^†^	10.8	139.91 ± 65.24	155.00	0.443 ^†^
Moderate	76.2	164.06 ± 74.67 ^a^	171.67		67.6	229.63 ± 54.00	237.50		82.3	125.35 ± 55.78	118.33	
Poor	6.1	150.93 ± 55.27 ^b^	163.33		5.0	189.72 ± 39.50	195.00		6.8	130.39 ± 51.95	120.00	
AJCC pathological stage												
I	31.8	173.39 ± 80.05	180.00	0.043 ^†^	35.2	235.16 ± 55.89	242.50	0.190 ^†^	29.3	120.08 ± 55.73	90.00	0.246 ^§^
II	33.4	161.89 ± 71.14	170.00		26.3	236.17 ± 45.71	237.50		38.6	125.53 ± 49.87	121.25	
III	15.2	157.81 ± 75.38	175.00		10.6	210.00 ± 61.10	195.00		18.5	136.25 ± 70.45	150.00	
IV	19.6	187.20 ± 69.41	190.00		27.9	223.35 ± 54.74	227.50		13.7	135.51 ± 55.17	130.00	
T classification												
T1	33.9	172.83 ± 79.29	180.00	0.044 ^†^	38.0	231.76 ± 58.05	242.50	0.196 ^†^	30.9	120.79 ± 55.60	90.00	0.585 ^†^
T2	43.7	166.91 ± 72.17	175.00		38.0	234.60 ± 47.46	232.50		47.8	128.23 ± 52.75	125.00	
T3	11.2	150.35 ± 74.88	170.00		6.7	200.42 ± 64.31	187.50		14.5	133.66 ± 71.30	127.50	
T4	11.2	192.19 ± 66.81	192.50		17.3	222.74 ± 52.21	230.00		6.8	136.47 ± 53.79	145.00	
N classification												
N0	77.3	167.84 ± 77.08	173.33	0.370 ^§^	74.9	232.56 ± 54.80	241.25	0.325 ^†^	79.1	123.82 ± 56.18	113.33	0.139 ^†^
N1	10.7	179.37 ± 62.37	185.00		12.3	215.80 ± 46.20	197.50		9.6	145.97 ± 56.82	165.00	
N2	11.9	174.66 ± 70.75	185.00		12.8	221.28 ± 55.16	225.00		11.2	135.54 ± 57.20	130.00	

Abbreviations: ^*^ *p* values were estimated by student’s *t*-test. ^†^ *p* values were estimated by one-way ANOVA test. ^§^ *p* values were estimated by Kruskal-Wallis one-way ANOVA test. ^a^ *p* < 0.001; ^b^ *p* = 0.011.

**Table 4:** Impact of ATG4B expression levels on Disease-specific survival (DSS) according to different demographic and clinicopathologic factors in patients with OSCC.

Variable	ATG4B	No. (%)	CHR (95% CI)	*p* Value *	AHR (95% CI)	*p* Value ^†^
Sex						
Female	Low	7 (20.6)	1.00		1.00	
	High	27 (79.4)	33.44 (0.08–13883.49)	0.254	290102.55 (0.00–7.435E + 285)	0.970 ^a^
Male	Low	65 (16.5)	1.00		1.00	
	High	329 (83.5)	1.52 (0.97–2.38)	0.067	1.74 (1.11–2.73)	0.016 ^a^
Age, yrs						
≤50	Low	32 (15.8)	1.00		1.00	
	High	170 (84.2)	2.10 (1.05–4.19)	0.035	1.57 (0.78–3.18)	0.209 ^a^
>50	Low	40 (17.7)	1.00		1.00	
	High	186 (82.3)	1.43 (0.79–2.57)	0.236	2.15 (1.17–3.92)	0.013 ^a^
Cell differentiation						
Well	Low	16 (21.1)	1.00		1.00	
	High	60 (78.9)	0.97 (0.32–2.99)	0.956	1.07 (0.34–3.30)	0.912 ^b^
Moderate, poor	Low	56 (15.9)	1.00		1.00	
	High	296 (84.1)	1.83 (1.12–3.00)	0.015	2.05 (1.26–3.36)	0.004 ^b^
AJCC pathological stage						
I, II	Low	47 (16.8)	1.00		1.00	
	High	232 (83.2)	1.41 (0.74–2.66)	0.294	1.37 (0.72–2.59)	0.335 ^c^
III, IV	Low	25 (16.8)	1.00		1.00	
	High	124 (83.2)	2.35 (1.25–4.40)	0.008	2.34 (1.25–4.40)	0.008 ^c^
T classification						
T1, T2	Low	53 (16.0)	1.00		1.00	
	High	279 (84.0)	1.39 (0.82–2.36)	0.219	1.26 (0.75–2.14)	0.384 ^d^
T3, T4	Low	19 (19.8)	1.00		1.00	
	High	77 (80.2)	3.21 (1.37–7.51)	0.007	3.10 (1.31–7.31)	0.010 ^d^
N classification						
N0	Low	61 (18.4)	1.00		1.00	
	High	270 (81.6)	1.44 (0.85–2.45)	0.179	1.68 (0.98–2.88)	0.060 ^e^
N1, N2	Low	11 (11.3)	1.00		1.00	
	High	86 (88.7)	2.07 (0.89–4.79)	0.090	1.95 (0.83–4.59)	0.124 ^e^
Postoperative RT						
No	Low	49 (15.8)	1.00		1.00	
	High	262 (84.2)	1.98 (1.06–3.70)	0.032	2.55 (1.36–4.79)	0.004 ^a^
Yes	Low	23 (19.7)	1.00		1.00	
	High	94 (80.3)	1.62 (0.85–3.10)	0.143	1.43 (0.74–2.75)	0.283 ^a^

Abbreviations: CHR, crude hazard ratio; CI, confidence interval; AHR, adjusted hazard ratio; RT, radiotherapy. * *p* values were estimated by Cox regression. ^†^ *p* values were estimated by multivariate Cox regression. ^a^ Adjusted for cell differentiation (moderate + poor vs. well) and AJCC pathological stage (stage III + IV vs. stage I + II). ^b^ Adjusted for AJCC pathological stage (stage III + IV vs. stage I + II). ^c^ Adjusted for cell differentiation (moderate + poor vs. well). ^d^ Adjusted for cell differentiation (moderate + poor vs. well) and N classification (N1, N2 vs. N0). ^e^ Adjusted for cell differentiation (moderate + poor vs. well) and T classification (T3, T4 vs. T1 + T2).

**Table 5:** Expression levels of ATG4B and pATG4B in relation to DSS of OSCC patients.

Variable		No. (%)	CHR (95% CI)	*p*-Value	AHR (95% CI)	*p*-Value
**OSCC**						
ATG4B	Low	72 (16.8)	1.00		1.00	
	High	356 (83.2)	1.70(1.09–2.66)	0.020	1.87(1.20–2.94)	0.006 *
Phospho-ATG4B	Low	44 (10.3)	1.00		1.00	
	High	384 (89.7)	1.13(0.69–1.87)	0.626	1.08(0.66–1.79)	0.752 *
ATG4B (L), pATG4B (L)		14 (3.3)	1		1	
Either		88 (20.6)	0.79 (0.54–1.16)	0.224	1.89 (0.58–6.18)	0.290 ^†^
ATG4B (H), pATG4B (H)		326 (76.2)	1.40 (0.97–2.02)	0.072	2.47 (0.79–7.75)	0.122 ^†^
**BMSCC**						
ATG4B	Low	28 (15.6)	1.00		1.00	
	High	151 (84.4)	1.72(0.82–3.57)	0.149	2.70(1.28–5.68)	0.009 *
Phospho-ATG4B	Low	6 (3.4)	1.00		1.00	
	High	173 (96.6)	1.30(0.32–5.28)	0.717	1.31(0.32–5.34)	0.708 *
ATG4B (L), pATG4B (L)		2 (1.1)	1		1	
Either		30 (16.8)	0.72 (0.37–1.39)	0.324	6201.44 (0.00–1.003E + 080)	0.922 ^†^
ATG4B (H), pATG4B (H)		147 (82.1)	1.53 (0.79–2.98)	0.210	8815.48 (0.00–1.425E + 080)	0.919 ^†^
**TSCC**						
ATG4B	Low	35 (14.1)	1.00		1.00	
	High	214 (85.9)	2.46(1.19–5.06)	0.015	2.19(1.06–4.52)	0.034 *
Phospho-ATG4B	Low	43 (17.3)	1.00		1.00	
	High	206 (82.7)	1.14(0.68–1.91)	0.630	1.07(0.64–1.81)	0.788 *
ATG4B (L), pATG4B (L)		14 (5.6)	1		1	
Either		50 (20.1)	0.81 (0.49–1.33)	0.401	1.96 (0.58–6.65)	0.278 ^†^
ATG4B (H), pATG4B (H)		185 (74.3)	1.48 (0.92–2.37)	0.104	2.57 (0.81–8.15)	0.109 ^†^

Abbreviations: * *p*-values were adjusted for cell differentiation (moderate + poor vs. well) and AJCC pathological stage (stage III + IV vs. stage I + II) by multiple Cox regression. ^†^ *p* values were estimated by multivariate Cox regression. Bold values denote statistical significance.

**Table 6:** Impact of pATG4B expression levels on DFS according to different demographic and clinicopathologic factors in patients with OSCC.

Variable	pATG4B	No. (%)	CHR (95% CI)	*p* Value *	AHR (95% CI)	*p* Value ^†^
Sex						
Female	Low	12 (35.3)	1.00		1.00	
	High	22 (64.7)	1.12 (0.28–4.47)	0.874	0.88 (0.21–3.58)	0.852 ^a^
Male	Low	67 (17.0)	1.00		1.00	
	High	327 (83.0)	1.33 (0.86–2.06)	0.206	1.44 (0.93–2.24)	0.105 ^a^
Age, yrs						
≤50	Low	38 (18.8)	1.00		1.00	
	High	164 (81.2)	1.63 (0.87–3.09)	0.130	1.74 (0.92–3.29)	0.089 ^a^
>50	Low	41 (18.1)	1.00		1.00	
	High	185 (81.9)	1.18 (0.68–2.04)	0.567	1.22 (0.70–2.13)	0.477 ^a^
Cell differentiation						
Well	Low	7 (9.2)	1.00		1.00	
	High	69 (90.8)	23.92 (0.04–14170.39)	0.330	540189.98 (0.00)	0.978 ^b^
Moderate, poor	Low	72 (20.5)	1.00		1.00	
	High	280 (79.5)	1.40 (0.92–2.13)	0.119	1.36 (0.89–2.07)	0.157 ^b^
AJCC pathological stage						
I, II	Low	57 (20.7)	1.00		1.00	
	High	222 (79.6)	1.04 (0.65–1.68)	0.863	1.15 (0.71–1.87)	0.558 ^c^
III, IV	Low	22 (14.8)	1.00		1.00	
	High	127 (85.2)	2.55 (1.03–6.34)	0.044	2.59 (1.04–6.45)	0.040 ^c^
T classification						
T1, T2	Low	64 (19.3)	1.00		1.00	
	High	268 (80.7)	1.21 (0.77–1.90)	0.402	1.30 (0.83–2.04)	0.260 ^d^
T3, T4	Low	15 (15.6)	1.00		1.00	
	High	81 (84.4)	2.49 (0.77–8.07)	0.129	2.29 (0.70–7.47)	0.170 ^d^
N classification						
N0	Low	67 (20.2)	1.00		1.00	
	High	264 (79.8)	1.13 (0.72–1.77)	0.605	1.24 (0.79–1.95)	0.354 ^e^
N1, N2	Low	12 (12.4)	1.00		1.00	
	High	85 (87.6)	2.69 (0.84–8.64)	0.097	2.59 (0.80–8.32)	0.112 ^e^
Postoperative RT						
No	Low	57 (18.3)	1.00		1.00	
	High	254 (81.7)	1.17 (0.73–1.86)	0.517	1.18 (0.74–1.90)	0.488 ^a^
Yes	Low	22 (18.8)	1.00		1.00	
	High	95 (81.2)	2.24 (0.89–5.65)	0.088	2.51 (0.99–6.34)	0.053 ^a^

Abbreviations: * *p* values were estimated by Cox regression. ^†^ *p* values were estimated by multivariate Cox regression. ^a^ Adjusted for cell differentiation (moderate + poor vs. well) and AJCC pathological stage (stage III + IV vs. stage I + II). ^b^ Adjusted for AJCC pathological stage (stage III + IV vs. stage I + II). ^c^ Adjusted for cell differentiation (moderate + poor vs. well). ^d^ Adjusted for cell differentiation (moderate + poor vs. well) and N classification (N1, N2 vs. N0). ^e^ Adjusted for cell differentiation (moderate + poor vs. well) and T classification (T3, T4 vs. T1 + T2).

**Table 7:** Expression levels of ATG4B and pATG4B in relation to DFS of OSCC patients.

Variable		No. (%)	CHR (95% CI)	*p*-Value	AHR (95% CI)	*p*-Value
**OSCC**						
ATG4B	Low	81 (18.9)	1.00		1.00	
	High	347 (81.1)	1.26(0.85–1.87)	0.245	1.22(0.82–1.81)	0.319 *
Phospho-ATG4B	Low	79 (18.5)	1.00		1.00	
	High	349 (81.5)	1.37(0.90–2.08)	0.137	1.45(0.96–2.21)	0.080 *
ATG4B (L), pATG4B (L)		20 (4.7)	1		1	
Either		120 (28.0)	0.80 (0.57–1.13)	0.119	1.29 (0.55–3.03)	0.555 ^†^
ATG4B (H), pATG4B (H)		288 (67.3)	1.33 (0.96–1.85)	0.085	1.67 (0.73–3.78)	0.222 ^†^
**BMSCC**						
ATG4B	Low	151 (84.4)	1.00		1.00	
	High	28 (15.6)	1.15(0.64–2.05)	0.640	1.19(0.66–2.13)	0.563 *
Phospho-ATG4B	Low	19 (10.6)	1.00		1.00	
	High	160 (89.4)	1.30(0.60–2.82)	0.514	1.38(0.63–3.00)	0.422 *
ATG4B (L), pATG4B (L)		19 (10.6)	1		1	
Either		132 (73.7)	1.02 (0.62–1.68)	0.936	1.27 (0.58–2.78)	0.552 ^†^
ATG4B (H), pATG4B (H)		28 (15.6)	1.15 (0.64–2.05)	0.640	1.42 (0.57–3.51)	0.453 ^†^
**TSCC**						
ATG4B	Low	42 (16.9)	1.00		1.00	
	High	207 (83.1)	1.77(0.97–3.23)	0.065	1.68(0.92–3.08)	0.095 *
Phospho-ATG4B	Low	38 (15.3)	1.00		1.00	
	High	211 (84.7)	1.93(1.00–3.71)	0.049	1.95(1.02–3.76)	0.045 *
ATG4B (L), pATG4B (L)		16 (6.4)	1		1	
Ether		48 (19.3)	0.58 (0.33–1.02)	0.059	1.21 (0.40–3.66)	0.741 ^†^
ATG4B (H), pATG4B (H)		185 (74.3)	1.91 (1.15–3.19)	0.013	2.20 (0.81–6.01)	0.123 ^†^

Abbreviations: * *p*-value were adjusted for cell differentiation (moderate + poor vs. well) and AJCC pathological stage (stage III + IV vs. stage I + II) by multivariate Cox regression. ^†^ *p* values were estimated by multivariate Cox regression. Bold values denote statistically significant differences.

## Supplementary Materials

The following are available online at https://www.mdpi.com/2072-6694/11/12/1854/s1 or s2, Figure S1. Validation of phospho-specific antibodies against phosphor-S383/392-ATG4B, Figure S2: Whole blots with densitometry readings and molecular weight ladders for Figure 5A,C.

## Appendix A

**Table A1:** Impact of pATG4B expression levels on disease-specific survival according to different demographic and clinicopathologic factors in patients with OSCC.

Variable	pATG4B	No. (%)	CHR (95% CI)	*p* Value *	AHR (95% CI)	*p* Value ^†^
Sex						
Female	Low	5 (14.7)	1.00		1.00
	High	29 (85.3)	1.43 (0.18–11.30)	0.735	1.09 (0.13–8.98)	0.939 ^a^
Male	Low	39 (9.9)	1.00		1.00	
	High	355 (90.1)	1.11 (0.66–1.85)	0.699	1.09 (0.65–1.83)	0.745 ^a^
Age, yrs						
≤50	Low	22 (10.9)	1.00		1.00	
	High	180 (89.1)	0.94 (0.49–1.81)	0.845	0.90 (0.47–1.74)	0.751 ^a^
>50	Low	22 (9.7)	1.00		1.00	
	High	204 (90.3)	1.38 (0.64–2.98)	0.417	1.27 (0.59–2.76)	0.544 ^a^
Cell differentiation						
Well	Low	5 (6.6)	1.00		1.00	
	High	71 (93.4)	1.16 (0.15–8.74)	0.887	1.88 (0.24–14.68)	0.548 ^b^
Moderate, poor	Low	39 (11.1)	1.00		1.00	
	High	313 (88.9)	1.21 (0.72–2.03)	0.466	1.05 (0.63–1.77)	0.846 ^b^
AJCC pathological stage						
I, II	Low	31 (11.1)	1.00		1.00	
	High	248 (88.9)	1.33 (0.61–2.89)	0.471	1.48 (0.68–3.23)	0.321 ^c^
III, IV	Low	13 (8.7)	1.00		1.00	
	High	136 (91.3)	0.88 (0.46–1.70)	0.705	0.83 (0.43–1.60)	0.580 ^c^
T classification						
T1, T2	Low	35 (10.5)	1.00		1.00	
	High	297 (89.5)	1.18 (0.64–2.20)	0.600	1.11 (0.60–2.08)	0.737 ^d^
T3, T4	Low	9 (9.4)	1.00		1.00	
	High	87 (90.6)	1.03 (0.44–2.40)	0.944	0.80 (0.33–1.90)	0.609 ^d^
N classification						
N0	Low	38 (11.5)	1.00		1.00	
	High	293 (88.5)	1.04 (0.57–1.90)	0.897	1.17 (0.64–2.13)	0.615 ^e^
N1, N2	Low	6 (6.2)	1.00		1.00	
	High	91 (93.8)	0.83 (0.33–2.06)	0.685	0.70 (0.27–1.80)	0.460 ^e^
Postoperative RT						
No	Low	30 (9.6)	1.00		1.00	
	High	281 (90.4)	1.20 (0.61–2.37)	0.603	1.07 (0.54–2.13)	0.846 ^a^
Yes	Low	14 (12.0)	1.00		1.00	
	High	103 (88.0)	1.23 (0.59–2.57)	0.584	1.19 (0.57–2.49)	0.651 ^a^

Abbreviations: * *p* values were estimated by Cox regression. ^†^ *p* values were estimated by multivariate Cox regression. ^a^ Adjusted for cell differentiation (moderate + poor vs. well) and AJCC pathological stage (stage III + IV vs stage I + II). ^b^ Adjusted for AJCC pathological stage (stage III + IV vs. stage I + II). ^c^ Adjusted for cell differentiation (moderate + poor vs. well). ^d^ Adjusted for cell differentiation (moderate + poor vs. well) and N classification (N1, N2 vs. N0). ^e^ Adjusted for cell differentiation (moderate + poor vs. well) and T classification (T3, T4 vs. T1 + T2).

**Table A2:** Impact of ATG4B expression levels on disease-free survival according to different demographic and clinicopathologic factors in patients with OSCC.

Variable	ATG4B	No. (%)	CHR (95% CI)	*p* Value *	AHR (95% CI)	*p* Value ^†^
Sex						
Female	Low	7 (20.6)	1.00		1.00	
	High	27 (79.4)	31.66 (0.05–20701.19)	0.296	188230.91 (0.00–1.249E+286)	0.971 ^a^
Male	Low	74 (18.8)	1.00		1.00	
	High	320 (81.2)	1.15 (0.78–1.71)	0.484	1.13 (0.76–1.67)	0.555 ^a^
Age, yrs						
≤50	Low	33 (16.3)	1.00		1.00	
	High	169 (83.7)	1.13 (0.63–2.05)	0.588	0.94 (0.51–1.73)	0.838 ^a^
>50	Low	48 (21.2)	1.00		1.00	
	High	178 (78.8)	1.39 (0.82–2.35)	0.221	1.57 (0.92–2.66)	0.096 ^a^
Cell differentiation						
Well	Low	19 (25.0)	1.00		1.00	
	High	57 (75.0)	0.93 (0.34–2.59)	0.893	0.94 (0.34–2.60)	0.898 ^b^
Moderate, poor	Low	62 (17.6)	1.00		1.00	
	High	290 (82.4)	1.26 (0.82–1.93)	0.290	1.27 (0.83–1.94)	0.282 ^b^
AJCC pathological stage						
I, II	Low	53 (19.0)	1.00		1.00	
	High	226 (81.0)	1.37 (0.82–2.30)	0.235	1.32 (0.79–2.22)	0.289 ^c^
III, IV	Low	28 (18.8)	1.00		1.00	
	High	121 (81.2)	1.12 (0.61–2.05)	0.712	1.07 (0.58–1.96)	0.836 ^c^
T classification						
T1, T2	Low	60 (18.1)	1.00		1.00	
	High	272 (81.9)	1.25 (0.80–1.98)	0.330	1.16 (0.74–1.84)	0.518 ^d^
T3, T4	Low	21 (21.9)	1.00		1.00	
	High	75 (78.1)	1.29 (0.59–2.81)	0.519	1.13 (0.51–2.50)	0.755 ^d^
N classification						
N0	Low	68 (20.5)	1.00		1.00	
	High	263 (79.5)	1.33 (0.84–2.11)	0.226	1.30 (0.82–2.06)	0.266
N1, N2	Low	13 (13.4)	1.00		1.00	
	High	84 (86.6)	0.88 (0.42–1.88)	0.748	0.78 (0.37–1.67)	0.524 ^e^
Postoperative RT						
No	Low	55 (17.7)	1.00		1.00	
	High	256 (82.3)	1.43 (0.87–2.36)	0.159	1.44 (0.88–2.38)	0.150 ^a^
Yes	Low	26 (22.2)	1.00		1.00	
	High	91 (77.8)	1.02 (0.53–1.96)	0.947	1.06 (0.55–2.07)	0.857 ^a^

Abbreviations: * *p* values were estimated by Cox regression. ^†^ *p* values were estimated by multivariate Cox regression. ^a^ Adjusted for cell differentiation (moderate + poor vs. well) and AJCC pathological stage (stage III + IV vs stage I + II). ^b^ Adjusted for AJCC pathological stage (stage III + IV vs stage I + II). ^c^ Adjusted for cell differentiation (moderate + poor vs. well). ^d^ Adjusted for cell differentiation (moderate + poor vs. well) and N classification (N1, N2 vs. N0). ^e^ Adjusted for cell differentiation (moderate + poor vs. well) and T classification (T3, T4 vs. T1 + T2). RT: radiation therapy.
