# Supplementary material for: Association of ATG4B and Phosphorylated ATG4B Proteins with Tumorigenesis and Prognosis in Oral Squamous Cell Carcinoma
Source: Cancers (Basel). 2019 Nov 23;11(12):1854. doi: 10.3390/cancers11121854 (PMC6966594; doi:10.3390/cancers11121854)
Supplement: Supplementary file 1 [file cancers-11-01854-s001.pdf]

Supplementary Materials: Association of ATG4B and Phosphorylated ATG4B Proteins with Tumorigenesis and Prognosis in Oral Squamous Cell Carcinoma

Pei-Feng Liu, Hung-Chih Chen, Jin-Shiung Cheng, Wei-Lun Tsai, Huai-Pao Le, Shu-Chi Wang, Wei-Hao Peng, Cheng-Hsin Lee, Luo-Ping Ger and Chih-Wen Shu

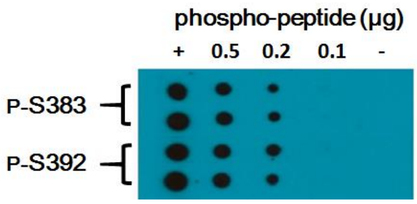

Figure S1. Validation of phospho-specific antibodies against phosphor-S383/392-ATG4B. The specificity of antibody against phosphor-S383/392 ATG4B were validated by Dot Blot assay using various concentration of phosphor-S383 and phosphor-S392 peptide, respectively (Figure S1). The normal rabbit serum reacted with secondary antibody is used as a positive control, whereas bovine serum albumin (BSA) can not react with secondary antibody and serves as a negative control.

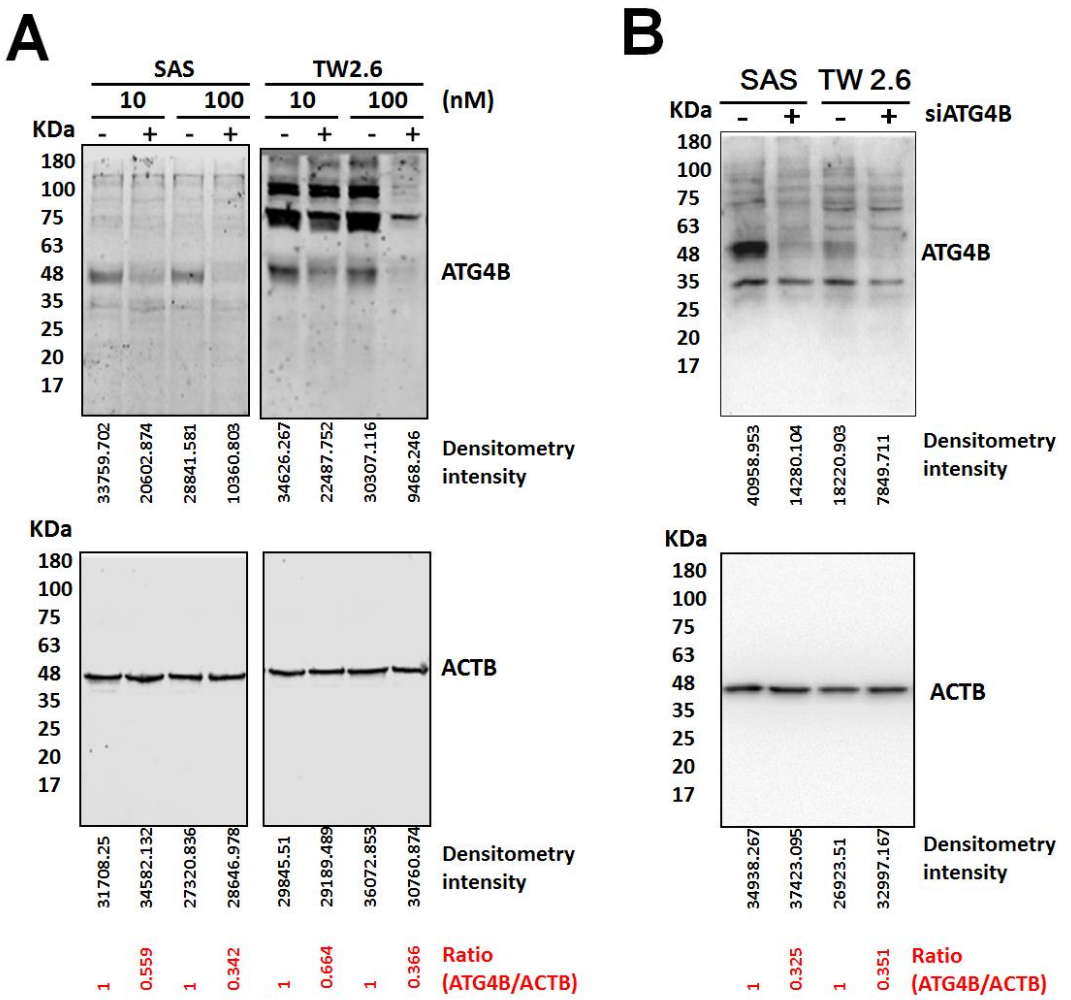

Figure S2. Whole blots with densitometry readings and molecular weight ladders for Figure 5A,C. (**A**) The whole blot is original membrane corresponding to Figure 5A. Briefly, the TSCC

cell line SAS and BMSCC cell line TW2.6 were transfected with scrambled (−) antisense oligonucleotide (ASO) or ASO against ATG4B (+) for 72 h, and the silencing effect was verified by immunoblotting. (**B**) The whole blot is original membrane corresponding to figure 5A. The OSCC cells were transfected with scramble siRNA (−, 5 nM) or siRNA against ATG4B (+, 5 nM) for 66 h. The knockdown efficiency was determined by immunoblotting.

© 2019 by the authors. Licensee MDPI, Basel, Switzerland. This article is an open access article distributed under the terms and conditions of the Creative Commons Attribution (CC BY) license (http://creativecommons.org/licenses/by/4.0/).
